# Reply to Dr. Kinglake on the Obstetric Practice

**Published:** 1816-08

**Authors:** J. Atkinson


*For the London Medical and Physical Journal.*

*Reply to Dr. Kinglake on the Obstetric Practice;* by J. Atkinson, Esq.

AFTER I had read Dr. Merriman's able defence of the present practice of midwifery, I thought the arguments and documents which he adduced so conclusive, as to render any farther encroachment upon your valuable pages unnecessary; but, as the subject is of the highest importance, as well to society as to those honorable practitioners who are engaged in the profession, I conceive myself warranted in making a few more remarks in order to remove a stigma, which I conceive has been unjustly thrown upon the art. Dr. Merriman has anticipated me in several arguments; but, if the following observations can illustrate and confirm more fully what he has said, as well as answer some of the objections which have escaped his notice, I shall consider myself amply remunerated.

Dr. Kinglake's attempt to prove, that the present practice of midwifery is not only unnecessary, but pernicious, has I think completely failed. He deprecates the custom of "boring the fœtal scull after waiting only twelve hours, and when the mother and attendants (forsooth) have had no apprehensions of danger." Now, when the pelvis is distorted, and the diameter from sacrum to pubis is not more than an inch and a half, how, I would ask, can so large a body as the head of a child pass through this cavity? Common sense is sufficient to determine even *à priori.* Nature would here exhaust all her resources in vain, as happened in the case mentioned in my last communication; where then, under these circumstances, would be the impropriety of perforating the head, when we know that such an expedient would ensure a safe delivery? while waiting would only augment the danger and prolong the pain, without any chance of benefit to the patient. I trust, however, and believe, notwithstanding the opprobrious insinuation of Dr. Kinglake, that no practitioner who values his character, would resort to this operation, until he is fully convinced of the inadequacy of all other means. How man-midwifery can be pernicious or dangerous, I am totally at a loss to conceive, for I apprehend that the danger to any patient will be in proportion to the ignorance or inhumanity of the practitioner, whether male or female; the question therefore is, whether a person who is intimately acquainted with the powers of nature, the laws of the animal economy, the aberrations of nature, as well as the most efficient method of obviating those aberrations, or or a person totally destitute of this knowledge, (which is the case with most ordinary midwives), I say, which of these is most likely to do harm? I could bring a long list of facts to solve this question, at which humanity would shudder, (for I have had the opportunity of seeing a good deal of their handy work in a few neighbouring villages), but this appears to me an unnecessary trespass upon your pages. If Dr. K. imagines that women do less than men in natural cases, I can assure him he is most egregiously mistaken; the fact is, that, where nothing ought to be attempted, they do a great deal, as in rupturing the membranes, extracting the placenta, &c. I heard of a midwife a little while ago, who, in order to expedite her patient's labour, shook her, by the assistance of some other persons, so violently that she became quite sore. The only instance wherein I can perceive any disadvantage to the public, resulting from the male practice of midwifery, is the expense attending it, (unless we mention the injury to their own health and comfort, and to the delicacy of the patient); but this I think scarcely deserves notice, if it can be ascertained (which it has most satisfactorily by Dr. Merriman) that many valuable lives are annually saved by it, especially when we consider that about five times as much is expended in the article of tobacco, and about ten times as much in tea; the one highly deleterious, and the other at least useless, not to mention innumerable other articles of luxury. If Dr. Kinglake, however, could succeed in convincing medical men of the superiority of women in ordinary midwifery cases, I am sure he would find no small difficulty in persuading females, and particularly those who have tried both.

If the sagacity of man is not required, in order "to regulate the motion of the celestial bodies, or to modify the universal principle of attraction," both totally out of his province, are we therefore to conclude, that he has no power to influence any operation of nature, not even that of relieving the distresses of his fellow creatures? Man is evidently endued with a considerable portion of that intelligence which seems to be every where exerted in creation for the promotion of happiness and perfection; in many instances his interference is absolutely necessary; and he seems to have been reserved, in the grand scheme of things, as an auxiliary agent, to complete the benevolent design: this is strikingly illustrated in many surgical operations, also in the healing of wounds, as well as in human parturition.

The popular opinion respecting midwifery is undoubtedly erroneous; so it is with regard to medicine,—the public often attribute to the physician that fame which belongs only to the *vis medicatrix naturæ;* indeed, where there is much ambiguity, I believe the popular opinion is generally wrong. But, although the good women imagine that in all cases the obstetrician is to afford actual manual assistance, and that he can, under any circumstances, deliver whenever he thinks proper; yet this affords no argument why he should therefore, in order that his practice may coincide with their prejudices, interfere with the salutary operations of nature; neither do I believe those operations, when efficient, ever are intermeddled with, unless by those practitioners who are destitute both of knowledge and humanity.

With regard to the successful labours of the Asiatic, the African, and the uncivilized American women, I would observe, in addition to Dr. Merriman's arguments,—First, that as correct tables of lying-in women in those parts have not been produced by any historian that I know of, we cannot form a proper estimate of their danger. Secondly, Mr. White, of Manchester, and Professor Camper, have fully demonstrated, that the structure of the pelvis, as well as that of the bones of the fore-arm and of the head, is very different in these women from that of the European. Mr. White says, (as near as I can recollect,) "that, in consequence of the approximation of the African scull to that of the monkey, not one of this tribe could ever be made to comprehend the problems in geometry. Thirdly, historians relate that many of these women plunge into a river soon after they are delivered, which would probably kill an European lady.*

If these facts are correctly stated, it appears, that the above circumstance can no more affect our arguments in favour of accoucheurs, than the parturition of brutes, which is daily occurring before our eyes.

Dr. Kinglake makes the two following assertions, which, if am not greatly mistaken, I shall be able to invalidate most completely.

1st, He asserts "that medical practitioners in *full midwifery employ* upwards of thirty years, have never met with an unnatural presentation, have never had an occasion for

* The Indians in the Isthmus of America receive no injury from plunging into cold water when in a sweat; and, as the most speedy remedy for intoxication, the women throw their husbands into a river when they are drunk. The minute after delivery, women scruple not to bathe in cold water with their infants, and yet, dangerous as we should consider this practice, these women are rarely known to die in child-bearing.—*Buffon's Nat. Hist.* page 345, vol. 3.

an an instrument, and have always found the natural efforts equal to all the exigencies of salutary parturition."

Secondly, He asserts, "that he believes not one practitioner in a thousand in any age has met with a case of placental presentation."—If assertions be considered any proof, however, I assert, and I speak from experience, that the converse is much nearer the truth (viz. that not one practitioner in a thousand, in full midwifery employ, has failed to meet with a case; I have met with three in the course of ten years' practice.

For the following statement, I am indebted to Mr. Hey, jun. who has been so obliging as to give me a brief description of every case here mentioned; they occurred successively in a given and not a long period of that gentleman's practice; it is but just to state, however, that to many of the cases (probably not less than thirty) he was called in consultation with other practitioners. From these documents it appears, that, out of 827 labors, 150 were such as to require manual aid, either with regard to the expulsion of the placenta or child.

5 were cases of presentation of the placenta.
9 cases of arm or shoulder presentation.
3 ——— hydrocephalus.
41 ——— breech and feet presentation.
3 ——— puerperal convulsions.
2 ——— ruptured uterus.
2 ——— arm presentation, in which turning was impracticable; but the delivery was effected by art in another manner.

The remainder were, face presentations, floodings, cases that required the extraction of the placenta, &c.

Surely Dr. Kinglake will be more cautious in future how he makes assertions so open to attack; for, were he espousing the cause of truth, and assailing some serious popular error, a recourse to falsehood, either through ignorance or design, would not be very likely to ensure success.

I have seen Dr. Kinglake's last communication in answer to Dr. Merriman, and have to remark, that it is principally an effusion of wit, exerted against the latter gentleman for raising a "hue and cry" about murder; this accusation seems to have stung the doctor so keenly, that it has engrossed his whole attention, insomuch that he has forgotten to attempt a confutation of the demonstrative evidence there brought forward of the superiority of accoucheurs; which evidence rests not upon vain speculation, as in the case of the ancient method of treating the small-pox and yellow fever, but upon the solid basis of fair, extensive, and reite-

 rated rated experiment, which can admit of no ambiguity, and on which the philosopher may as safely rely, as he may upon the demonstrations of Euclid.

I think whoever has candidly examined the present discussion must perceive that the doctor has completely failed in establishing several of his positions, and that we are warranted in concluding—

First, that preternatural cases are much more frequent and dangerous than he has asserted. Secondly, that women, as they are at present educated, are much more mischievous, intermeddling, and inefficient, than accoucheurs. Now, if these propositions are demonstrated, this corollary irresistibly follows, viz. that, although we cannot deny, that many midwifery cases would terminate favorably, if left solely to the operations of nature, yet, in a state of civilization (if not in a savage state) the cases of danger and wrong presentation are so numerous, as to demand the regular attendance of conscientious and well-educated men; that the practice is not one of those instances of pernicious craft, which time has rendered sacred, but a necessary and salutary aid which can do no harm when properly exercised, but which has saved the life of thousands, and which, if not as extensively useful, is as certainly so as vaccination.

*Leeds;*
*June* 24, 1816.

